# A comprehensive study on the microstructure evolution and oxidation resistance of conventional and nanocrystalline MCrAlY coatings

**DOI:** 10.1038/s41598-020-79323-w

**Published:** 2021-01-13

**Authors:** Farzin Ghadami, Alireza Sabour Rouh Aghdam, Soheil Ghadami

**Affiliations:** Department of Materials Engineering, Tarbiat Modares University, Tehran, 14115-143 Iran

**Keywords:** Energy science and technology, Engineering, Materials science, Nanoscience and technology

## Abstract

Conventional and nanocrystalline MCrAlY coatings were applied by the high-velocity oxy-fuel (HVOF) deposition process. The ball-milling method was used to prepare the nanocrystalline MCrAlY powder feedstock. The microstructure examinations of the conventional and nanocrystalline powders and coatings were performed using X-ray diffraction (XRD), high-resolution field emission scanning electron microscope (FESEM) equipped with energy-dispersive X-ray spectroscopy (EDX), transmission electron microscope (TEM), and X-ray photoelectron spectroscopy (XPS). Williamson–Hall analyzing method was also used for estimation of the crystalline size and lattice strain of the as-milled powders and sprayed coatings. Owing to the investigation of the oxidation behavior, the freestanding coatings were subjected to isothermal and cyclic oxidation testing at 1000 and 1100 °C under static air. The results showed that the conventional as-sprayed MCrAlY coating had a parabolic behavior in the early stage and prolonged oxidation process. On the contrary, in the case of the nanocrystalline MCrAlY coating, the long-term oxidation behavior has deviated from parabolic to sub-parabolic rate law. Moreover, the results also exemplified that the nanocrystalline MCrAlY coating had a greater oxidation resistance following the creation of a continuous and slow-growing Al_2_O_3_ scale with a fine-grained structure. The nucleation and growth mechanisms of the oxides formed on the nanocrystalline coating have also been discussed in detail.

## Introduction

Nowadays, research and technological studies focused on the enhancement of high-temperature materials and coatings which standing in high-temperature corrosion and oxidation damages have been increased^1–7^. Among with the variety of high-temperature coatings, MCrAlY coatings (M = Ni, Co, or NiCo) are widely used in some critical parts especially for gas turbine and aero-engine applications, where they may be applied as oxidation and hot corrosion resistant overlays or as bond-coats for use with thermal barrier coatings (TBCs). The addition of Ni in the coating composition cause to improving its oxidation resistance. Also, the presence of Co in the coating can increase the hot corrosion resistance and high-temperature fatigue life. A small percentage of active elements, such as Y is beneficial for MCrAlY coatings owing to increasing oxide scale uniformity and adhesion during high-temperature service^1,8^.

In the case of aero-engines and land-based turbines, it is well elucidated that the high-temperature oxidation hot corrosion stability of those industrial MCrAlY coatings depends on two main factors including their composition and microstructure. The characteristics of the feedstock powder, the coating deposition method, and possible post-heat treatments (e.g. vacuum annealing) are primary factors that determine the overall microstructure and composition of the developed coating.

Besides, the preparation technology of the feedstock powder, overall density, and microstructure of the coating as well as post-deposition treatments have a significant effect on the thermal stability and oxidation behavior of the MCrAlY coatings^9,10^. In most cases, the oxidation kinetics of MCrAlY coatings is mainly diffusion-controlled and the oxide growth rate follows a parabolic behavior^10–17^. There are limited studies on the deviation from parabolic behavior for the oxidized Ni-based and MCrAlY coatings for both cases of single overlay or bond coat for thermal barrier coatings (TBC)^18–21^. Generally, it has been reported that any deviation from parabolic rate law may be attributed to some parameters such as the partial pressure of the oxygen in the oxidizing atmosphere, rare of in-service heating and cooling, overall design of the part surface, composition and structural properties of the coatings and initiation of the microcracks in the coating structure^7,20,22^. For specific cases, such as the thermal stability of the nanocrystalline Ni-based coatings, the description of the oxidation mechanism is still limited, and presented explanations are more complicated^23^.

Thermal spraying technology is recently utilized to develop depositions with ultrafine/nanocrystalline structure. Among different deposition techniques to obtain nanocrystalline coatings, the HVOF spraying process with lower deposition temperature and super-sonic flame speed was mainly preferred to other thermal spray deposition techniques such as APS and VPS/LPPS methods. In the case of the HVOF deposition process, the as-sprayed coating layer indicates a minor microstructural change with a reasonable structural density during coating build-up^24^. Simultaneously, owing to the higher cooling rate of the melted or partially melted droplets on the surface, rapidly-solidified regions or the ultrafine-grained/nano-scaled structure may appear in the as-sprayed coating. The nano-scaled structure of the sprayed layer was almost arising from the structure of the as-milled powder^25,26^.

The isothermal and cyclic thermal stability of the MCrAlY coatings is mainly ascribed to its susceptibility to form a continuous and dense Al_2_O_3_ scale when subjected to the high-temperature service conditions^10^. In general, the durability of the overlay MCrAlY coating is still related to the homogeneity, structural density, and adhesion of the oxide scale formed on the coating. Among them, rapid-growing oxides including Ni or Cr containing oxides and spinels can increase the thermal stresses in the interface between coating and oxide scale; which may lead to promote oxide spallation from the surface^13,16,23,27–30^.

Mercier et al.^23^ developed nanocrystalline NiCoCrAlY deposits by the optimized cryomilling method. They estimated a crystalline size of about 15 nm for the as-sprayed NiCoCrAlY coating developed from 16 h cryomilled powder feedstock. They also indicated that an adherent oxide scale without any signs of delamination and perpendicular cracks was performed on the nanocrystalline coating after the oxidation test at 1000 °C for 48 h. The formation of a dense Al_2_O_3_ scale with higher adhesion can increase the high-temperature resistance of the nanocrystalline MCrAlY coating. In another investigation, Ma et al.^31^ prepared the NiCrAlY bond coating for the TBC system using the cryomilling process. They indicated that the nanostructured NiCrAlY bond-coat has relatively better oxidation stability owing to the creation of a protective and Al_2_O_3_ oxide scale together with the nucleation and growth of the dispersed Al- and Y-rich oxide phase with higher thermal stability into the structure of NiCrAlY coating.

In the present study, the state of the art on the preparation and characterization of the nanocrystalline MCrAlY coatings has been evaluated. Indeed, the novelty of the current work is to evaluate the structural properties and oxidation behavior by means of the calculated crystalline size for the developed nanocrystalline MCrAlY coating. Thus, the nanocrystalline MCrAlY feedstock powder was developed by the milling process. Subsequently, the conventional and nanocrystalline MCrAlY depositions were also applied using the HVOF spraying process. Structural features and thermal stability of the conventional and nanocrystalline MCrAlY coatings under isothermal and cyclic conditions have been characterized and the obtained results have been discussed in detail. Finally, the growth mechanism of the oxide scale formed on the nanocrystalline coating has also been discussed in detail.

## Experimental procedures

### Materials and powder preparation

The commercial MCrAlY powder studied in this work was a Ni–21Cr–9Al–0.8Y (wt%) with a product code Amdry 9624 (Oerlikon-Metco, Westbury, USA) deposited on low-carbon steel plates (0.12 wt% C) substrate. The original powder composition in wt% and characteristics are presented in Table 1.

**Table 1:** The composition and characteristics of the Amdry 9624 MCrAlY powder.

Powder formulation	Ni-21Cr-9Al-0.8Y (wt%)
Powder size	− 37 + 11 (µm)
Manufacturing process	Gas atomized
Powder morphology	Spheroidal

For the preparation of nanocrystalline MCrAlY powder, the milling process was performed in a PM-200, Retsch, Germany attrition mill up to 15 h with a ball to powder ratio of 30:1. Hard chrome-plated stainless steel balls, 0.5 cm in diameter, were as the grinding media. The milling cups were filled with Ar to prevent unwanted powder oxidation during milling. The stearic acid was also used as a process control agent (PCA) at 0.4 wt%, in order to avoid excessive agglomeration of the processed powder and to facilitate the breakage of the particles. The major process parameters of the milling were listed in Table 2.

**Table 2:** Process parameters of ball-milling to obtain nanocrystalline MCrAlY feedstock powder.

Parameter	Value
Milling medium	Ar atmosphere
Periodic rest time	10 min h^−1^
PCA	Stearic acid (0.4 wt%)
Rotational speed	300 rpm
Ball-to-powder ratio	30:1
Milling duration	5, 10 and 15 h

### HVOF spraying process

The conventional and nanocrystalline coatings were prepared using a commercial-grade of HVOF coating equipment (Hypojet 2700, MEC, Metallizing Equipment Co. PVT. Ltd, India) working with oxygen and propane (C_3_H_8_) system. The average value of the thickness of the coating was 250 ± 50 μm for all cases of the conventional and nanocrystalline coatings. The processed powders were then sieved to achieve the optimum particle size distribution for HVOF spraying.

In the current study, the surface of substrates was polished with a fine emery paper (number 800) to prepare a smooth surface before the spraying. Regarding obtained results from our previous studies^22,32–34^, the air-cooled HVOF torch was used for the spraying process. The spraying parameters are summarized in Table 3. After HVOF deposition, owing to the preparation of the freestanding coatings, the coated plates were bent around a cylindrical mandrel until the freestanding coating was detached from the substrate.

**Table 3:** HVOF spraying parameters for the as-received and nanocrystalline MCrAlY coatings.

Parameters	Conventional coating	Nanocrystalline coating
Fuel type	C_3_H_8_	C_3_H_8_
Fuel flow rate (l min^−1^)	55	55
Oxygen flow rate (l min^−1^)	210	200
Powder feeder rotation (rpm)	400	430
Powder feed rate (g min^−1^)	38	35
Carrier gas type	N_2_	N_2_
Carrier gas flow rate (l min^−1^)	20	22
Stand-off distance (mm)	250	250
Compressed air pressure (bar)	10	10
Traverse speed (mm rev^−1^)	60	60
Pass spacing (mm)	15	15
Number of cycles	6	8

### Structural characterizations

The morphology of the processed powder and microstructure of the obtained coatings were analyzed by a transmission electron microscope (TEM, Phillips, CM300, Netherlands), field emission electron microscope (FESEM, MIRA3-TESCAN, Czech Republic) equipped with energy-dispersive X-ray spectroscopy (EDX, INCA, Oxford Instruments). The preparation process of the freestanding coating specimens for structural observation was carried out using the procedure described in our previous works^22,35,36^. For the investigations of the structural phases, the conventional and nanocrystalline MCrAlY powders were characterized by the X-ray Diffraction test (XRD) with Cu-Kα radiation (λ = 1.54056 Å) at 40 kV and 40 mA (X’pert, Phillips, Netherlands). The obtained diffraction spectra of the coatings were then indexed and verified according to the International Centre for Diffraction Data (ICDD).

Measurements of the microhardness of the conventional and nanocrystalline coatings were carried out using a Vickers microhardness instrument (Micromet-1, Buehler, USA) with a test load of 300 g for 15 s. The standard deviation is based on the average value for five measurements made on each type of coatings. The area percentage of the structural porosities and β-phase content in the coatings were obtained using the image analyzing method (Image-J software, NIMH, ver. 1.2, USA). In this case, at least five structural image micrographs with the same magnification (1000×) were performed for the image analyzing process for all conventional and nanocrystalline coatings. Estimation of the crystalline size and lattice strain of the nanostructured powders under various milling durations as well as their developed coatings were carried out by examining the overall width of the diffraction peaks obtained from XRD patterns for the conventional and nanocrystalline powders and coatings using the analysis based on Williamson–Hall analysis 0. According to the Williamson–Hall method (Eq. 1), estimated values of the crystalline size (D_c_, nm) and lattice strain percentage (η, %) can be obtained by^37^:

1 $$\beta\;cos\;(\theta )= \frac{k\lambda }{{D}_{c}}+ \eta\;sin\;(\theta )$$

where β is the full width at half maximum (FWHM) value of the diffraction peak (radians), θ represents the diffraction angle (radians), k is the shape factor (~ 0.9) and A represents the wavelength of the X-ray source (Cu-Ka, nm).

### Isothermal and cyclic oxidation evaluations

In order to evaluate the high-temperature oxidation behavior of the conventional and nanocrystalline MCrAlYs, rectangular freestanding coatings (5 × 10 mm) were subjected to oxidation testing. Also, to obtain similar testing conditions, all the freestanding coating surfaces and edges were subsequently polished using 1000 grit paper (Ra ≈ 0.11 μm). The average thickness of all specimens before the oxidation tests was about 200 μm. The freestanding samples were then subjected to isothermal and cyclic oxidation tests at 1000 and 1100 °C respectively in laboratory air. For the case of the isothermal oxidation testing, two modes of short- and long-term oxidation tests were conducted under different oxidation times (up to 500 h for short-term and 1000 h for long-term tests). In addition, for the case of the thermal cycling test, each cycle included 1 h heating and 15 min subsequent cooling, and also the total cycles were 300 for all types of coatings.

For the case of the isothermal oxidation, the oxidation kinetics was evaluated from the measured weight gain ∆m (mg) and isothermal oxidation duration (t)^22^:

2 $$\left(\frac{\Delta m}{A}\right)=K{t}^{n}+B$$

where K is the oxidation rate constant (g cm^−2^ s^−n^), n represents oxidation law index (n ≈ 0.5 for parabolic rate oxidation behavior^38^) and B is a constant. Moreover, the structural studies of the oxide scale formed on the freestanding conventional and nanocrystalline MCrAlY coatings were conducted by FESEM and EDX. In addition, the X-ray photoelectron spectroscopy (XPS, PHI Quantera SXM, USA) was performed to investigate the surface chemistry of the oxidized coating.

## Results and discussion

### Characterization of the nanocrystalline powders

**Figure 1:**
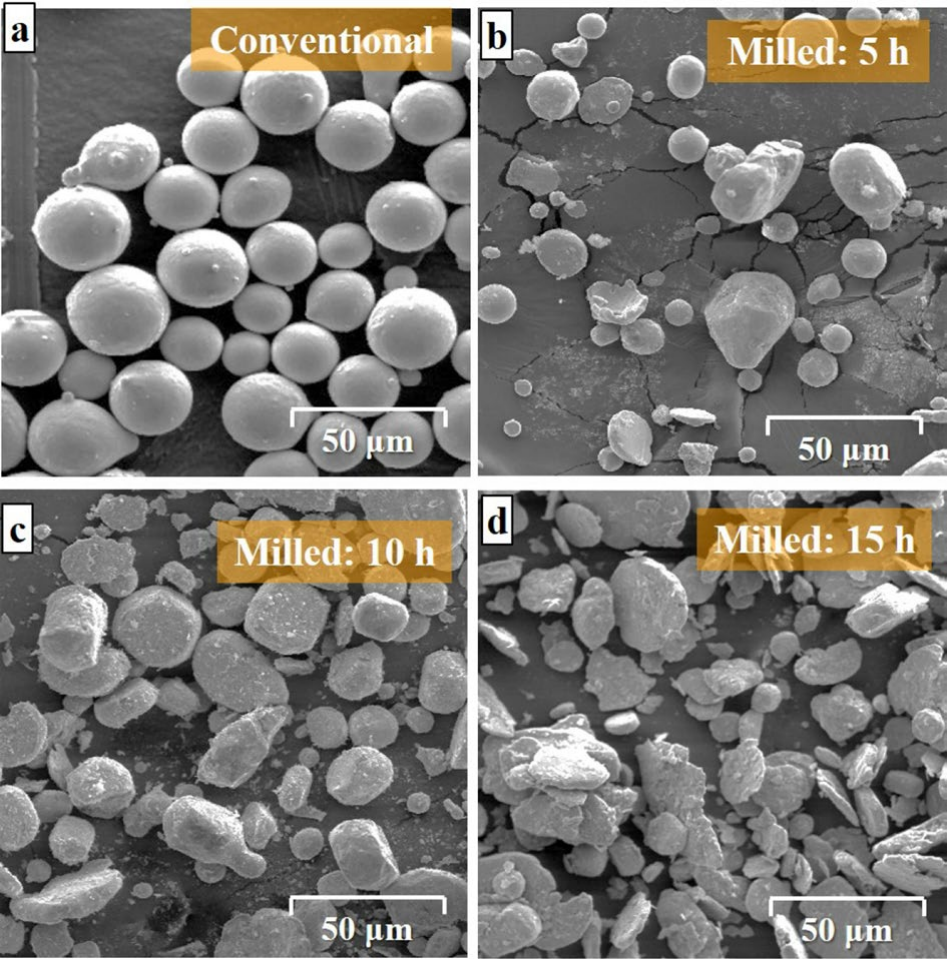
FESEM images of the morphology of the (**a**) commercial MCrAlY and as-milled MCrAlY powders after (**b**) 5 h, (**c**) 10 h and (**d**) 15 h.

Figure 1 represents the morphological FESEM images of the commercial MCrAlT (Fig. 1a) as well as processed MCrAlY powders after 5 h (Fig. 1b), 10 h (Fig. 1c), and 15 h (Fig. 1d) of the mechanical milling under Ar-controlled atmosphere. Moreover, when looked at in more detail, it is obvious that the as-received commercial MCrAlY powder shows spherical-shaped morphology with a smooth surface, which is the typical result of its gas atomization preparation process. Conversely, as can be observed, the as-milled MCrAlY powder exhibits deformed particles with a disk-shaped morphology. This morphology arises from the severe plastic deformation (SPD) process due to the ball and powder collisions during the milling process.

The value of collision-induced stresses was simultaneously increased with elongating the ball-milling process. At this middle stage of the milling, the morphology of the MCrAlY particles was changed to flaky-shaped morphology due to the SPD process^39^. By continuing the ball/powder and wall/powder collisions, the agglomerated morphology was obtained for the milled powder at the final stages of the milling after 15 h due to the consecutive breakage and cold welding process^23,40,41^. As can be observed from Fig. 1d, the morphology of the 15 h milled powder was transformed into a crashed particles with a flaky shape.

**Figure 2:**
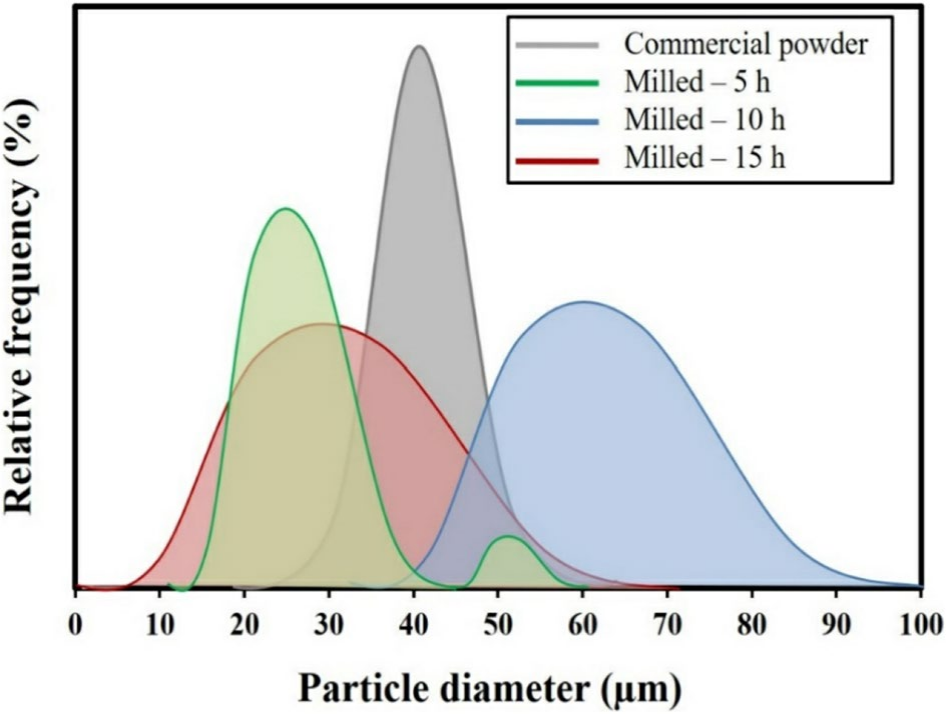
Particle size distribution of the commercial as well as milled MCrAlY powders with different milling durations.

Figure 2 shows the calculated particle size distribution of the commercial and milled MCrAlY powder using image analyzer software. As can be seen, the mean particle size of the commercial powder was between 35 and 45 μm after 15 h of the milling process, the mean particle size of the nanocrystalline powder was between 20 and 35 μm. Achievement of smaller particle size for nanocrystalline powder is related to higher breakage of processed powder upon a prolonged milling process. More details about the effect of milling time on particle size distribution on MCrAlY are available in our previous works^22,37^.

According to our previous study^37^, with increasing the milling time, at a specified strain level, a nanometric structure including low-angle grain boundaries and dislocation networks has emerged. Meanwhile, following increasing the milling these low-angle grain boundaries were transformed into the random-oriented nanostructures. Thus, by increasing milling time the crystalline size of the powder was progressively decreased until the final stage of milling (15 h) that the saturated particle size for the powder mixture was achieved^22,40,42,43^.

**Figure 3:**
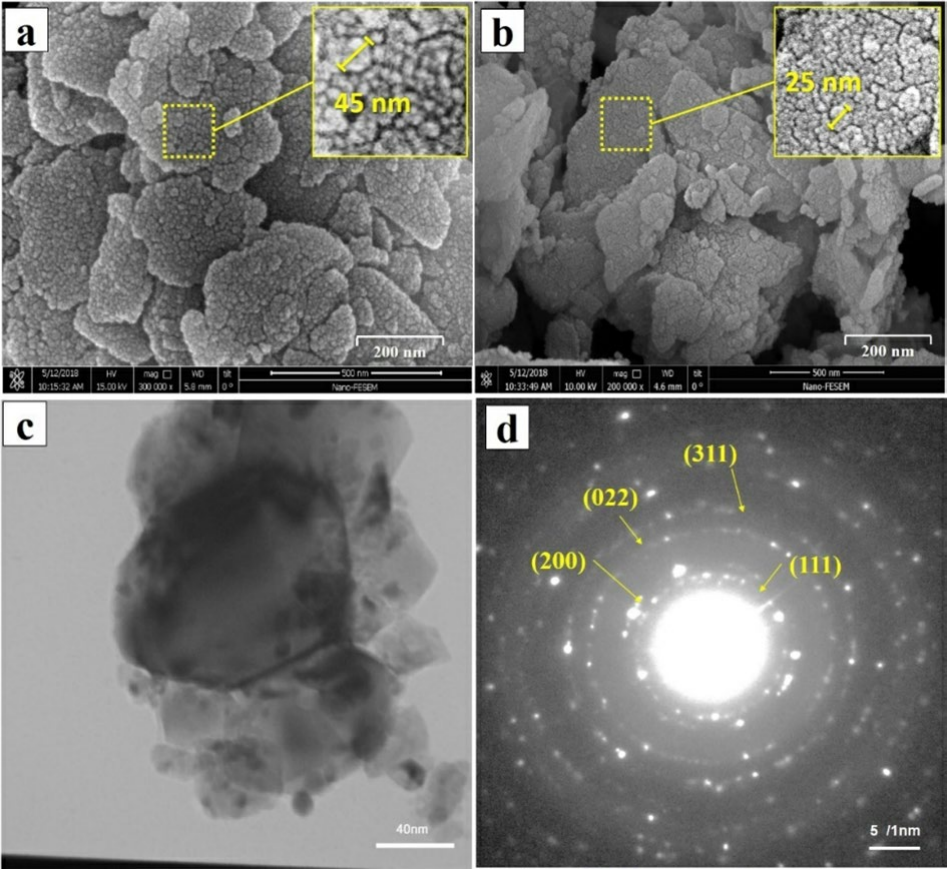
(**a**) FESEM images (secondary electron) from the surface of the nanocrystalline powder after 10 and (**b**) 15 h of ball-milling process, (**c**) TEM bright-field image of a nanocrystalline powder particle developed after 15 h of the milling process, and (**d**) corresponding SAD pattern represented nanocrystalline Ni-based γ phase in the milled powder particle.

Figure 3 shows the high-magnification structure of the as-milled MCrAlY powder achieved by FESEM and TEM observations after 10 and 15 h of the ball-milling process. Regarding Fig. 3a, the milling process leads to the development of nanocrystalline MCrAlY powder including the numerous agglomerates (~ 41 nm) of nanoparticles. By elongating the milling process, the average size of each nanoparticle was reduced to ~ 25 nm (Fig. 3b). For better comparison, Fig. 3c indicated the bright-field (BF) TEM images of the nanocrystalline MCrAlY powder obtained after 15 h of the milling. As can be seen, each powder particle consisted of numerous deformed and agglomerated MCrAlY nanoparticles. In accordance with the corresponding selective area diffraction (SAD) pattern indicated in Fig. 3d, the major diffraction rings were indexed as (111), (200), (022), and (311) planes which represented a Ni (γ) phase matrix in nanocrystalline powder. Moreover, broader rings in the SAD pattern may be attributed to the formation of a nanostructured MCrAlY powder after 15 h of the milling process.

Indeed, during the middle and the last stages of the milling, the value of the average particle size of MCrAlY powder was accordingly decreased owing to the incessant particle breakage of large particles. Moreover, the prolonged milling process may lead to increase particle breakage and reduce the particle crystallite size to the creation of an ultrafine-grained/nanocrystalline structure in the milled MCrAlY powder.

**Figure 4:**
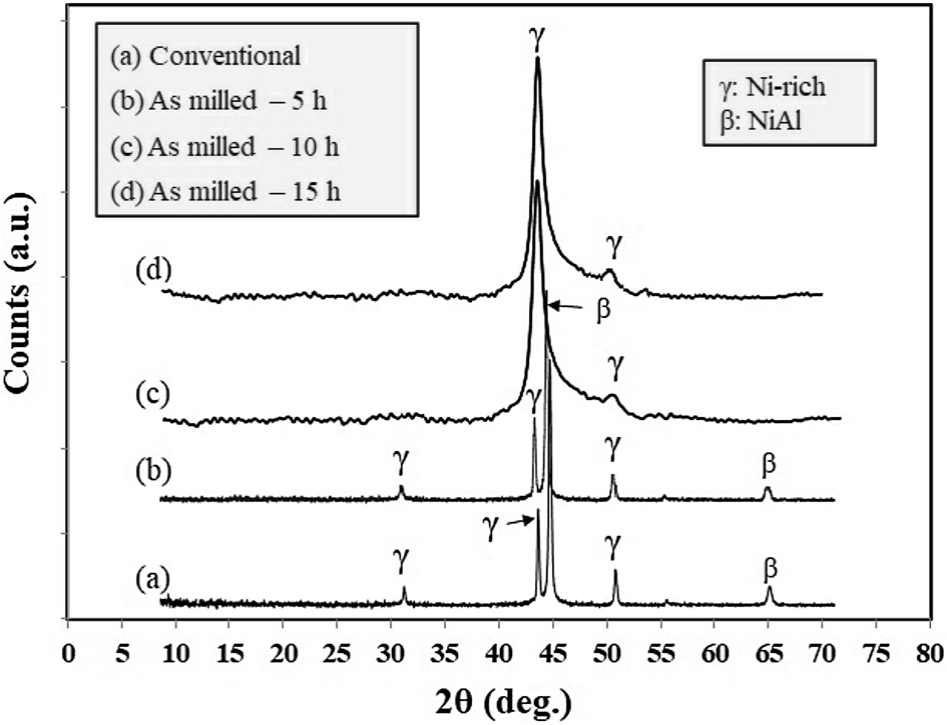
X-ray diffraction patterns of the commercial and mechanical-milled MCrAlY powders after 5, 10, and 15 h.

Figure 4 showed the X-ray diffraction spectra of the as-received and ball-milled MCrAlY powders under various milling times up to 15 h. As can be observed, in ball-milled powders higher than 10 h, β-phase precipitates disappeared due to the SPD process resulting in powder/wall and powder/balls during the high energy ball milling process. These findings are in agreement with other literature^40,44,45^.

**Figure 5:**
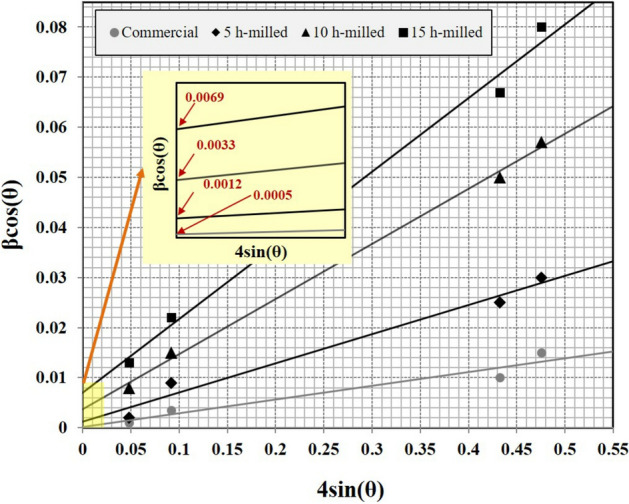
Williamson–Hall plots derived from the X-ray diffraction spectra of the commercial and ball-milled MCrAlY powders under different milling times.

Figure 5 indicated the linear Williamson–Hall plots obtained from the XRD spectra for the as-milled MCrAlY powders (Fig. 4). As can be seen, the crystal size of the as-milled particles can be calculated from the Y-intercept of the linear trendlines of the β·cos (θ) − 4 sin (θ) plot. Additionally, as can be observed from Williamson–Hall results, a lower slope of the linear trendline in the commercial MCrAlY powder exemplified the lower percentage of the lattice strain. In contrast, the milled MCrAlY powders have a relatively higher trendline slope indicating higher amounts of lattice strain because of their deformation during milling. Additionally, as can be seen, the trendline slopes of the milled powder were increased by elongating the milling process. Other Williamson–Hall investigations on the CoNiCrAlY powders have supported these findings^33,40^.

**Figure 6:**
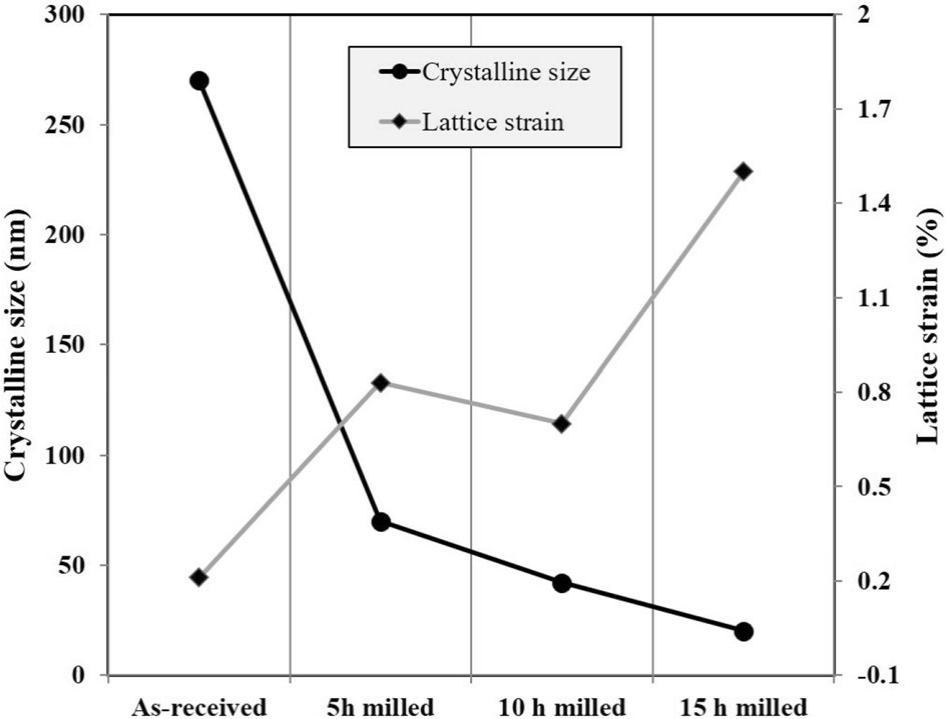
Variations of values of crystallite size and lattice strain percentage of Ni-rich γ-phase in the commercial and nanocrystalline MCrAlY powders as a function of milling duration.

The calculations of the crystalline size and lattice strain percentage for the as-received and ball-milled MCrAlY powder were carried out using the Williamson–Hall method (Eq. 1). The estimations were presented in Fig. 6. At the beginning of the milling, because of particle deformation and initial breakage, the lattice strain percentage was estimated about 0.25%. Besides, the crystalline size of the powder mixture was accordingly reduced from 270 to 115 nm. Afterward, the crystalline size of the milled MCrAlY powder was progressively decreased from 47 to 28 nm by increasing milling time from 10 to 15 h. At the last stages of the ball-milling, the lattice strain percentage significantly improved up to 1.3%. These crystalline estimations are in accordance with the results obtained from the structural evaluations of the milled MCrAlY powder (see Fig. 3b). It is worth noting that, in the following results related to coatings, due to minimum crystalline size and suitable morphology of the milled powder after 15 h, structural characteristics and oxidation resistance of the coating was focused on the nanocrystalline MCrAlY coating obtained from the 15 h milled powder and the results were compared with that conventional coating.

### Investigation of the nanocrystalline coatings

**Figure 7:**
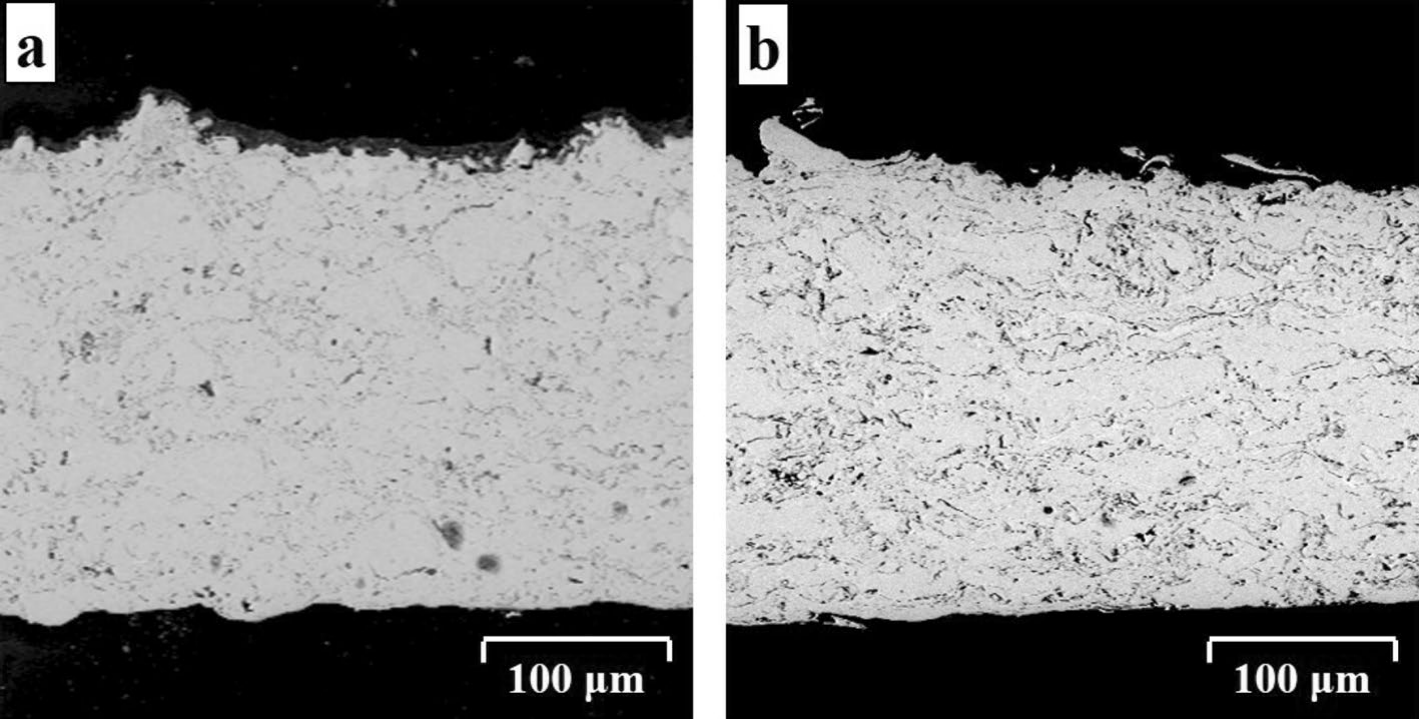
Backscattered electron images of (**a**) as-received MCrAlY coating and (**b**) nanocrystalline MCrAlY coatings developed using powder feedstock after 15 h of ball-milling process.

Backscattered electron micrographs of the as-sprayed conventional and nanocrystalline MCrAlY deposits obtained from the milled powder feedstock after 15 h are presented in Fig. 7. According to Fig. 7a, a coarse lamellar morphology with fewer amounts of porosity and oxides as well as the un-melted regions have been detected in conventional MCrAlY coating. Furthermore, the structure of conventional MCrAlY coating indicates two typical phases consisting of the Ni-based solid solution (γ) and NiAl (β). The image analyzing estimation for the as-sprayed MCrAlY coating indicates that the overall porosity content was about 1.6 ± 0.3%.

Conversely, a typical lamellar structure with finer morphology consisting of porosity and oxides were detected in the nanocrystalline MCrAlY coatings obtained by milled powders after 15 h according to Fig. 7b. Except for the conventional MCrAlY, a relatively dense structure with a little increase of calculated porosity (2.3 ± 0.1%) can be observed in the nanocrystalline coating after 15 h of the milling process. According to Fig. 7a, the bulky commercial powders cannot spread and flatten extensively, so the little amounts of structural porosities and oxides remain inside the conventional MCrAlY coating. Conversely, due to the preparation of nanocrystalline powders, by elongating the milling process, the tendency of the formation of agglomerated and nanostructured particles was accordingly increased, and hence, the probability of particle flattering to form a lamellar structure with suitable structural density.

Besides that, following the partial heating/melting process for some commercial MCrAlY particles during spraying, relatively coarser lamellae and splats were formed (Fig. 7a). Similar to the findings obtained by Daroonparvar et al.^46^ for MCrAlY coatings, the inter-splat pores, and microcrack was also distinguished in the FESEM microstructure of the conventional MCrAlY coating after thermal cycling test.

**Figure 8:**
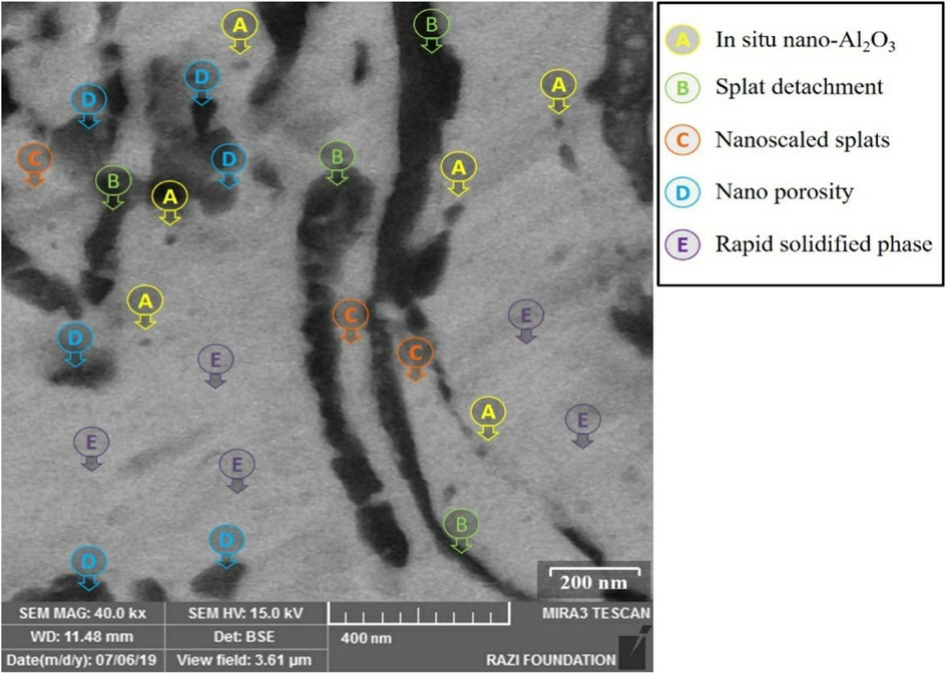
High-resolution FESEM micrograph of the splat formation and detailed structure of the nanocrystalline MCrAlY coating obtained from milled powder after 15 h (backscatter electron mode).

High-resolution FESEM image of the as-deposited nanocrystalline coating obtained by milled powder after 15 h was presented in Fig. 8. As can be observed, owing to the adequate melting of agglomerated (15 h milled) powder during spraying deposition, a finer nanometric structure consisting of nano-splats (C) and their boundaries (B) with scattered nano-scaled oxides (A) and porosities (D) was detected from the FESEM image. In addition, because of using nanocrystalline powder, the coating with nanoscaled structure was accordingly developed after the HVOF spraying^20,47^. Simultaneously, following the rapid solidification of heated or partially-melted particles during spraying, the ultrafine-grained/nanoscaled structure (E) may form in the coating structure. As mentioned earlier, the structural features of the developed coatings were probably arising from the morphology and structure of their feedstock powder. It is noted that the well-flattened splats, as well as the higher structural density, can be detected in the FESEM image of the nanocrystalline MCrAlY coating developed by 15 h milled powder.

In another point of view, the 15 h milled powder has a disk-shaped morphology with a higher surface to volume ratio (see Fig. 1d). Therefore, they are probably more easily heated and subjected to melting by the HVOF jet compared with the commercial spheroidal MCrAlY powder. Unmelted splats from the milled powders retain their original nanostructure, whereas melted particles might develop a nanostructure due to their fast quenching. Indeed, the milled powder is finer than the commercial one, which confirms that the milled particles can be melted more easily and quenched more effectively during coating build-up owing to its smaller size and lower heat capacity. In addition, more splats and more oxide stringers can form in the nanocrystalline coatings obtained from the ball-milled powders after 15 h.

**Figure 9:**
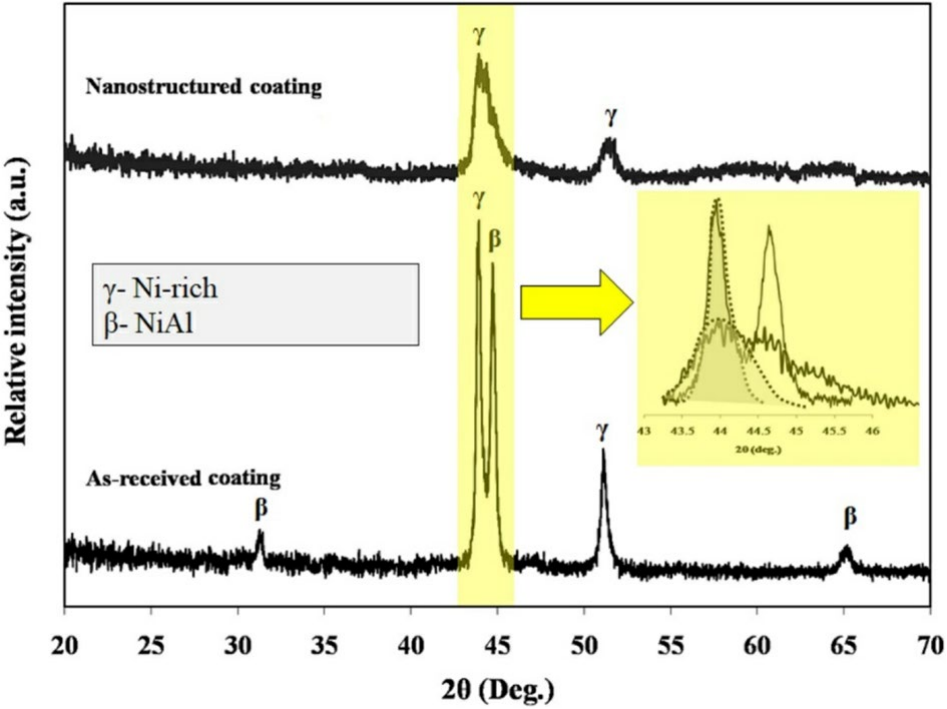
X-ray diffraction patterns of the as-received and nanocrystalline MCrAlY coatings applied by the HVOF spraying process.

Figure 9 shows the X-ray diffraction spectra of the conventional and nanocrystalline MCrAlY coatings. As can be seen, the diffraction spectra of the conventional MCrAlY coating shows two major Ni-rich (γ) and NiAl (β) phases. While the super-saturated γ-phase is only detected in the nanocrystalline MCrAlY layer. Furthermore, the γ-phase spectra for the nanocrystalline coating are broader than the related spectra for the conventional coating. This broadening effect might be attributed to the formation of the ultrafine-grained or nano-scale structure into the coating structure. In addition, owing to the increment of the structural/lattice strain fields and SPD of powder particles, the dissolution of β-phase can perform after the mechanical milling process^42^. Additionally, rapid solidification of in-flight particles during the spraying may another purpose for the peak broadening effect. This statement is supported by investigations carried out by Horita et al.^44^, Ghadami et al.^22,35^, and Bakker et al.^45^. In another point of view, the formation of the nanocrystalline coating may be raised not only from that its initial nanopowder but also can be maintained even after the HVOF spraying to the higher particle speed shorter time-of-flight. Similar findings were also presented in our previous works^22,35^ and also other studies by Mercier et al.^23^ and Saeedi et al.^28^.

**Figure 10:**
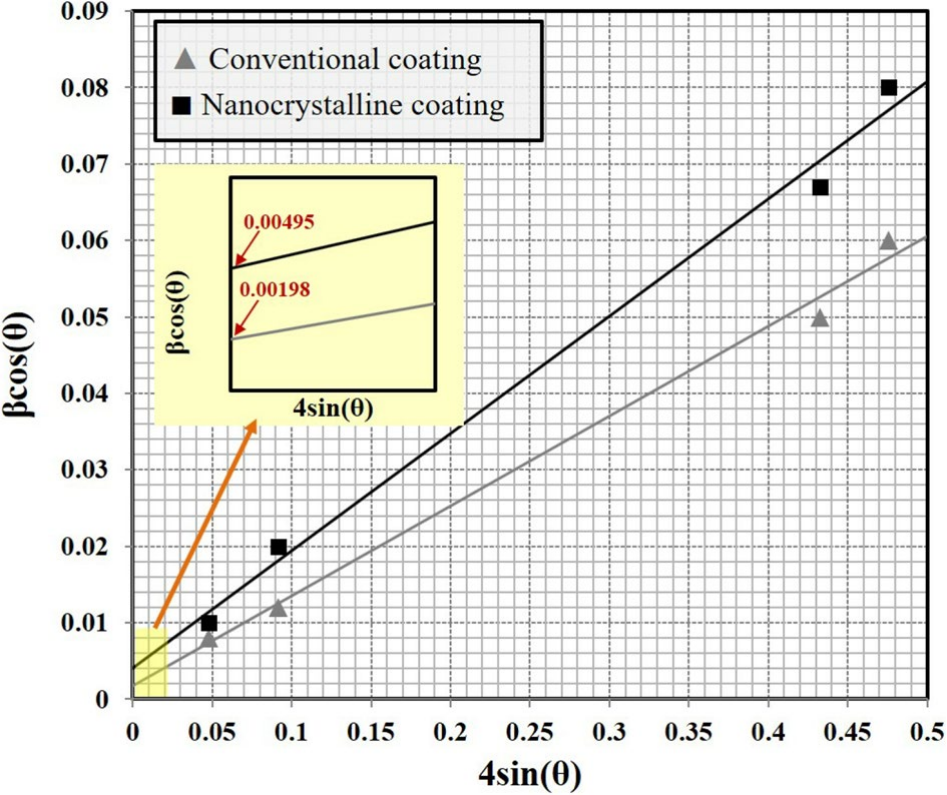
Williamson–Hall plots calculated for the conventional and nanocrystalline MCrAlY coatings deposited by the HVOF spraying technique.

Figure 10 shows the Williamson–Hall (cos θ vs. 4sin θ) plot which had derived from X-ray spectra of the conventional and nanocrystalline MCrAlY coatings. For this purpose, the magnified view of the main diffraction peaks are also provided for better estimation of the width of these two peaks (see Fig. 9). In addition, for precise calculation, the main peaks are specified by dashed line for two types of conventional and nanocrystalline coatings.

As can be observed from the plot, a lower value of the trendline slope for the conventional MCrAlY coating probably demonstrated the minimum percentage of the lattice strain. Conversely, the nanocrystalline MCrAlY coating has a relatively higher amount of the trendline slope. Further relevant studies based on the examinations of the crystalline size using Williamson–Hall estimations have supported this fact^48,49^.

**Figure 11:**
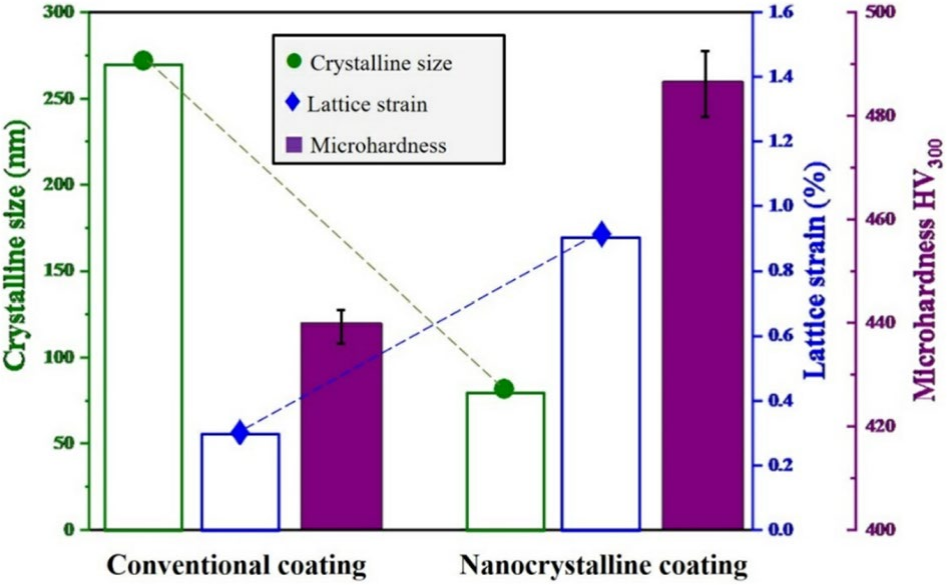
The comparative values of lattice strain and crystallite size estimated by the Williamson–Hall method as well as examined microhardness values of the conventional and nanocrystalline MCrAlY coatings.

Figure 11 indicates the estimated amounts of the lattice strain and crystallite size as well as the average microhardness values for both conventional and nanocrystalline MCrAlY coatings. As can be observed, the conventional coating has a lower amount of lattice strain compared to the nanocrystalline coatings. This can be related to the coarser lamellar microstructure of the conventional MCrAlY coating, which may also cause to indicate a lower microhardness. Also, the lattice strain percentage mainly arises from the ultra-high solidification process during the formation of splat on the surface. Differently, higher values of average microhardness and lattice strain have been obtained for the nanocrystalline MCrAlY coating. Indeed, the residual stress associated with the milling, impact/deformation of in-flight particles to form splats as well as maintaining the nanoscaled regime arisen from the as-milled nanopowder are the main causes of the improvement of the value of lattice stress/strain into the nanocrystalline MCrAlY coating. The mentioned results are supported by the microstructural results for the nanocrystalline MCrAlY coating deposited by 15 milled powder feedstock (Fig. 7b), which indicated a finer structural morphology and greater microhardness (485 ± 15 HV_300_) compared with the conventional MCrAlY coating (440 ± 15 HV_300_).

### Oxidation measurements of the coatings

#### Isothermal oxidation test

**Figure 12:**
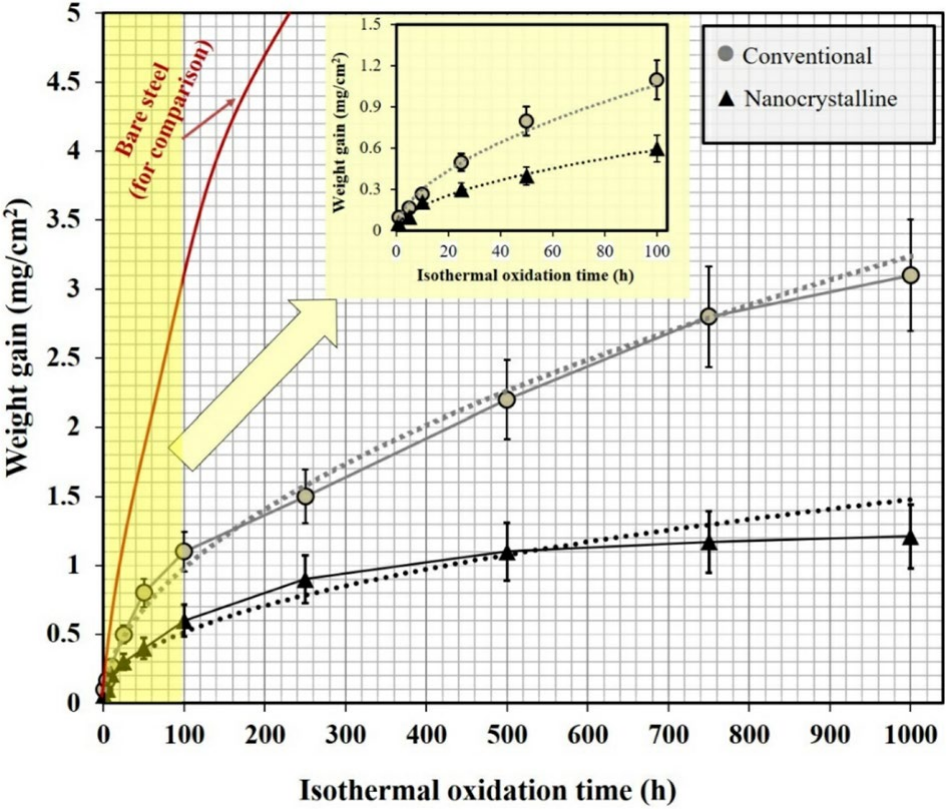
Average weight gain against isothermal oxidation time under long-term oxidation for the conventional and nanocrystalline MCrAlY coatings (15 h milled under 300 rpm) at 1100 °C.

Figure 12 indicated the specific weight gain versus exposure time for the conventional and nanocrystalline MCrAlY freestanding coatings at 1000 °C. As can be seen, the oxidation testing has been conducted in two duration steps. The first step is called short-term exposure (t < 100 h), and the second step is called long-term oxidation (100 < t < 1000 h). In the following, the parameters of the “n” index which specify the oxidation trend as well as oxidation rate constant (K) that defined oxidation behavior for both conventional and nanocrystalline MCrAlY coatings under short- and long-term oxidation test at 1000 °C were then carefully determined by Eqs. 2 and 3. Table 4 lists the comparative K and n parameters for the oxidized conventional and nanocrystalline coatings under two modes of short- and long-term oxidation examination.

**Table 4:** Oxidation rate constants (K) and “n” indexes of the as-received and nanocrystalline MCrAlY coatings under short- and long-term oxidation tests.

Coating type	Short-term exposure (0 < t < 100 h)			Long-term exposure (100 < t < 1000 h)		
	“n” index	Oxidation rate constant: K (g cm^−2^ s^−n^)	R^2^	“n” index	Oxidation rate constant: K (g cm^−2^ s^−n^)	R^2^
Conventional	0.47	1.66 × 10^–11^	0.95	0.52	1.89 × 10^–11^	0.96
	Oxidation behavior: Parabolic rate law			Oxidation behavior: Parabolic rate law		
Nanocrystalline	0.49	7.19 × 10^–12^	0.91	0.35	4.12 × 10^–12^	0.99
	Oxidation behavior: Parabolic rate law			Oxidation behavior: Sub-parabolic rate law		

For the short-term exposure results, both conventional and nanocrystalline coatings had a parabolic oxidation behavior at 1000 °C up to 100 h (n = 0.47 and 0.49, respectively). Moreover, the value of oxidation rate constant is minimal (K = 7.19 × 10^–12^ g cm^−2^ s^−n^) for the nanocrystalline MCrAlY coating compared with that conventional coating (K = 1.66 × 10^–11^ g cm^−2^ s^−n^). The obtained results emphasized that a better oxidation resistance under a similar exposing atmosphere (static air) at 1000 °C was also achieved for the nanocrystalline MCrAlY coating (about 31% higher than conventional MCrAlY coating).

It should be noted that the short-term oxidation behavior of both conventional and nanocrystalline MCrAlY coatings follows a parabolic law (“n” indexes in Table 4). Furthermore, the obtained results also demonstrated that the correspondence power trend can satisfactorily be fitted on short-term exposure results (“n” index almost near 0.5) for both conventional and nanocrystalline depositions at 1000 °C that confirm parabolic rate law for the exposure duration up to 100 h. Thus, due to the parabolic state of the conventional and nanocrystalline coatings, the oxidation behavior of them is mainly controlled by the diffusion process. The same parabolic rate behavior was also obtained in our previous work based on nano-CeO_2_ modified MCrAlY coatings^35^ and also other results obtained for the modified MCrAlY coatings elsewhere^23,50^.

In another point of view, the microhardness may has an indirect effect on the thermal properties (oxidation resistance) of the coatings. Indeed, with the increase of volume fraction of structural defects such as grain boundaries in the coating the value of microhardness was accordingly increased. Thus, as can be observed from microhardness data (Fig. 11), the higher percentage of grain boundaries in the coating structure may increase the tendency of internal oxidation of nanocrystalline coating at the early stages of the oxidation process at 1000 °C.

The obtained findings arise from the long-term oxidation data (Fig. 12) and calculations based on oxidation kinetics (see Table 4), show that the conventional MCrAlY coating still has parabolic behavior up to 1000 h of the exposure duration (n = 0.52). Differently, for the nanocrystalline MCrAlY coatings, the deviation from the parabolic to sub-parabolic rate law has been obtained (n = 0.35). Indeed, during prolonged oxidation testing, a significant reduction of the average weight gain was attained for the oxidized nanocrystalline MCrAlY coatings. Deviation from parabolic behavior may be related to the control or inhibition of the diffusion process by the formation of slow-growing and dense α-Al_2_O_3_ rather than other transient oxides and spinels^51^. The deviation from parabolic rate law for the nanostructured MCrAlY coatings has also been reported elsewhere^23^. The formation of Al_2_O_3_ prior to other oxides can effectively inhibit the outward diffusion of metallic ions during the prolonged oxidation process. Therefore, as predicted for the long-term exposure testing, the nanocrystalline coating followed a sub-parabolic rate law, whereas the oxidation rate constant of the nanocrystalline coating was still lower (K = 4.12 × 10^–12^ g cm^−2^ s^−n^) than that of the oxidation rate constant for the conventional MCrAlY coating (K = 1.89 × 10^–11^ g cm^−2^ s^−n^) in long-term exposure time.

It is worth noting that the creation and thickening of the α-Al_2_O_3_ oxide scale in the short-term exposure can perform by the inward diffusion of atomic oxygen and diffusion of Al-ions to the layers beneath the surface^52,53^. Indeed, In the early stages of the high-temperature exposure (up to 5 h), for the nanocrystalline MCrAlY coating, the formation of the predominant Al_2_O_3_ layer on the surface was susceptible owing to its finer grain size as well as higher volume ratio of grain boundaries to promoting the nucleation zones for Al_2_O_3_ oxide scale.

**Figure 13:**
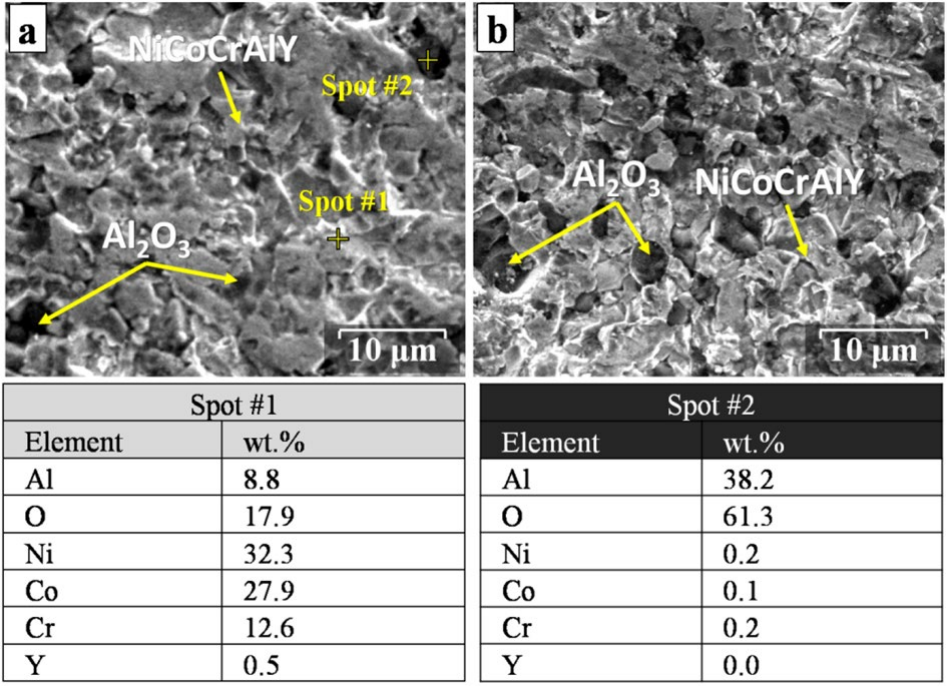
Secondary electron images of the surface of (**a**) as-received and (**b**) nanocrystalline MCrAlY coatings at the early stage of the isothermal oxidation process (5 h) at 1000 °C. EDX results from indicated points are also provided at the bottom of the images.

Figure 13 shows the secondary electron images of the surface of the oxidized coatings at the early stages of the isothermal oxidation (5 h) at 1000 °C. As can be seen from Fig. 13a, a lower amount of Al_2_O_3_ (black spots according to EDX results of spot#2) were appeared due to its coarser structure and limited nucleation sites for the formation of Al_2_O_3_ spots on the metallic surface of the MCrAlY coating (spot#1). Conversely, for the nanocrystalline coating, relatively higher amounts of Al_2_O_3_ oxide spots were detected due to the finer microstructure and higher amount of surface grain boundaries for nanocrystalline MCrAlY coating. Indeed, in the early stages of the oxidation, a relatively uniform and protective Al_2_O_3_ scale can form owing to the higher number of oxide spots on the surface. The formation of Al_2_O_3_ can provide a higher oxidation resistance in both short- and long-term oxidation process due to controlling the oxygen interdiffusion from the surface^20,54,55^.

**Figure 14:**
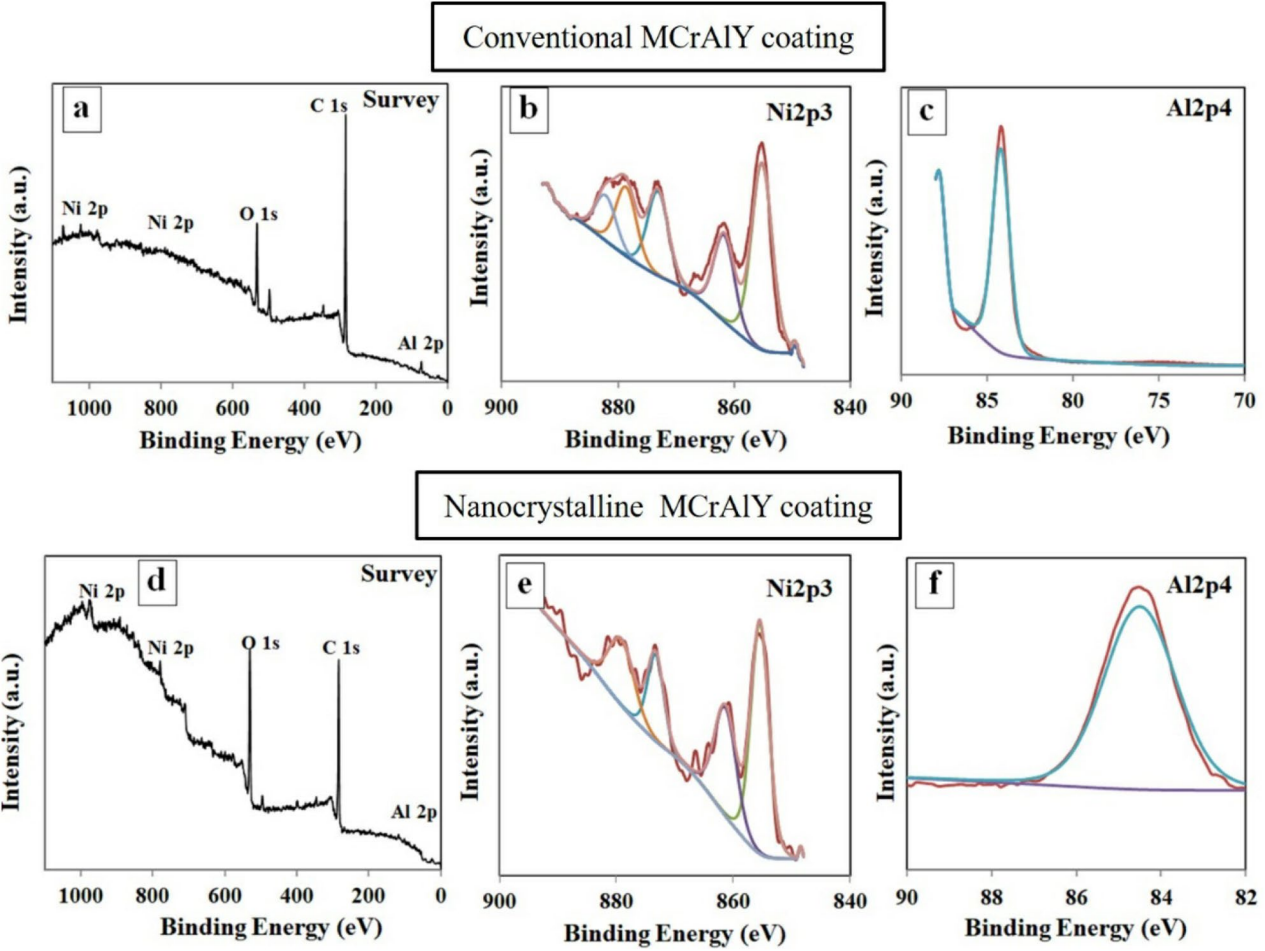
XPS results of the oxidized surface of the conventional and nanocrystalline coating: (**a**, **d**) survey peaks, (**b**, **e**) Ni 2p peaks, (**c**, **f**) Al 2p peaks.

Figure 14 shows the XPS results of the surface of the oxidized coatings at the early stages of the oxidation (5 h) at 1000 °C. As can be seen, in the uppermost layer of both types of oxidized coatings (Fig. 14a,d), oxygen and a much stronger signal of carbon originate from the residue gases, such as CO, CO_2,_ and O_2_, in the chamber formed during early stages of the oxidation. Oxygen is also adsorbed on the surface to form Al_2_O_3_ oxide phases. As can be observed, the higher intensity of O peaks for the nanocrystalline indicated that the value of the Al_2_O_3_ formed on the coating surface was higher than that of the conventional coating. In another point of view, mechanical milling can cause to increase C, N, and O impurities during milling and handling of the powder. In this case, the addition of stearic acid as PCA also likely further increased the C content in the powder mixture.

For the case of conventional coating, fast-growing oxides (such as NiO) can easily form at the early stages of oxidation up to 5 h compared with nanocrystalline coatings. The high-resolution spectra of Ni 2p for both types of coatings mainly belong to NiO monoxide (see Fig. 14b,e). In addition, the intensity of Ni-ions (which referred to Ni^2+^) is higher than that of the nanocrystalline coating. Conversely, the higher intensity of Al 2p (which belongs to Al_2_O_3_ oxide) for the conventional coatings is related to the higher growth rate of the alumina layer compared with the nanocrystalline coating (see Fig. 14c,f). In another point of view, the thermodynamically favored Al_2_O_3_ is formed and rapidly covered the uppermost of the coating surface during the early stages of the oxidation of the nanocrystalline coating. It must be noted that, in the early stages of oxidation, a relatively uniform and protective Al_2_O_3_ scale can form owing to the higher number of oxide spots on the surface. The formation of a uniform and thin Al_2_O_3_ scale can provide a higher oxidation resistance in the early stages of the oxidation due to controlling the oxygen interdiffusion from the surface^8,10^.

**Figure 15:**
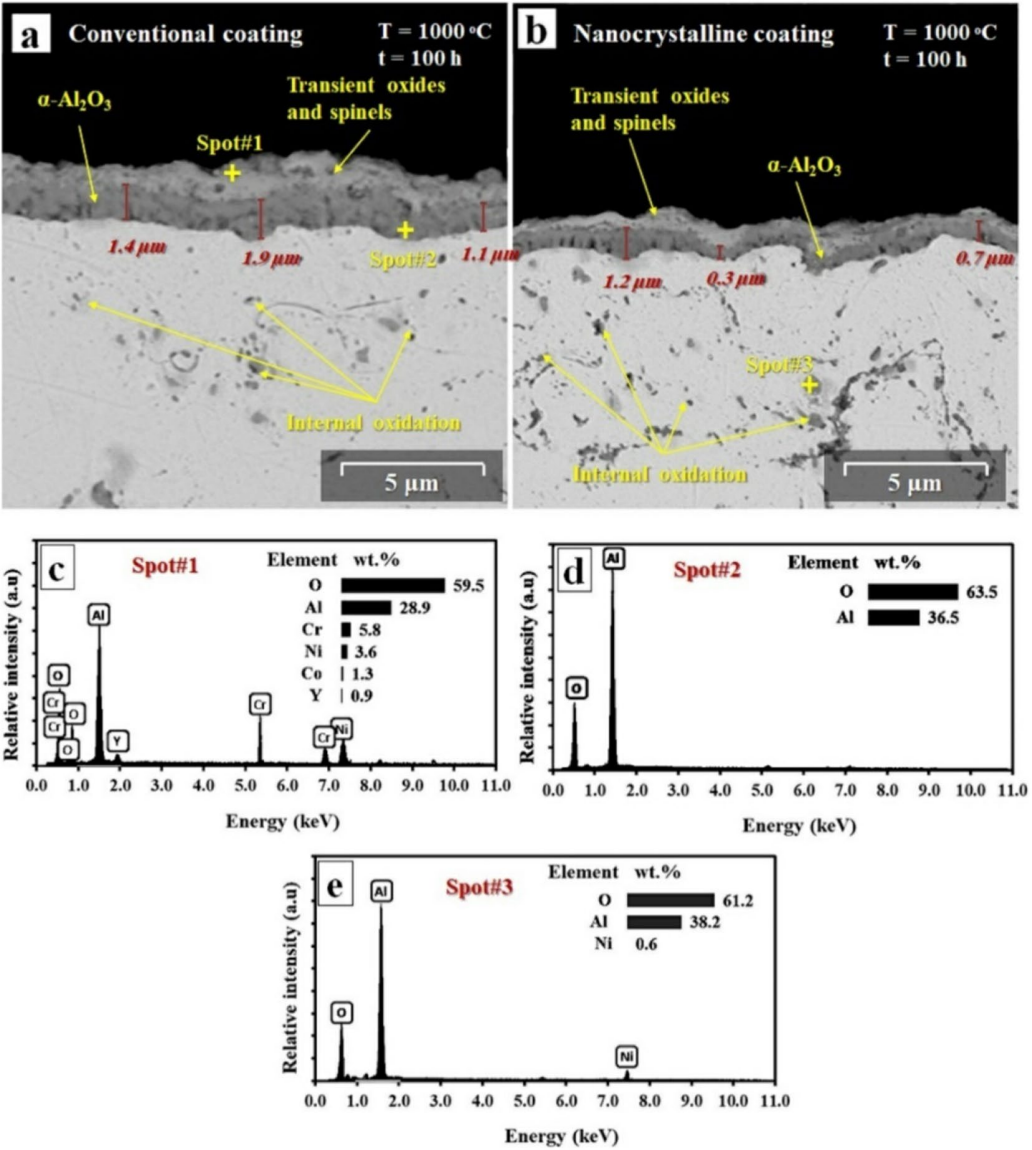
Backscattered FESEM images of the (**a**) conventional MCrAlY and (**b**) nanocrystalline MCrAlY coatings after 100 h of the short-term exposure at 1000 °C, (**c**–**e**) EDX results of the specified spots for the oxidized coatings.

Cross-sectional images of the oxidized conventional and nanocrystalline MCrAlY coatings under short-term oxidation at 1000 °C after 100 h as well as EDX analysis of oxide layers were presented in Fig. 15. As can be seen, the composition of the oxide layer in the conventional coating (Fig. 15a) is almost similar to the oxide layer in the nanocrystalline coating (Fig. 15b). Besides, as can be observed from the EDX results from spot #1 and referring to our previous investigation^26^, the overall composition of the oxide layers formed involved the upper-layer transient/mixed oxide, including NiO monoxide and NiAl_2_O_4_ spinel (Fig. 15c). Also, a relatively dense Al_2_O_3_ layer with darker contrast (spot #2) was also distinguished in the inner-layer for both conventional and nanocrystalline MCrAlY coatings (Fig. 15d). It is worth mentioning that the formation of internal oxide regions (spot #3) was also detected for both types of coatings after 100 h of oxidation.

As can be seen from the structure of the oxidized coatings, the relative area percentage of the internal oxide regions for nanocrystalline MCrAlY coating is relatively higher than that of the conventional coating. This higher internal oxide percentage is attributed to the finer structure and higher volume ratio of grain boundaries in the nanocrystalline MCrAlY coating.

As mentioned earlier, the high energy milling process can produce nanopowders with semispherical and agglomerated morphology including fine particles. This nanometric particles in each agglomerate are susceptible to absorb more oxygen from the atmosphere during the HVOF spraying process compared to the commercial spherical MCrAlY powder. The absorbed supersaturated oxygen can maintain in the γ phase in the nanocrystalline MCrAlY coating^28,46^. Thus, a higher percentage of the internal oxide regions were formed into the oxidized nanocrystalline coating owing to the high-temperature chemical reaction of soluble oxygen and Al ions in the first stages of the oxidation process^20,22^.

**Figure 16:**
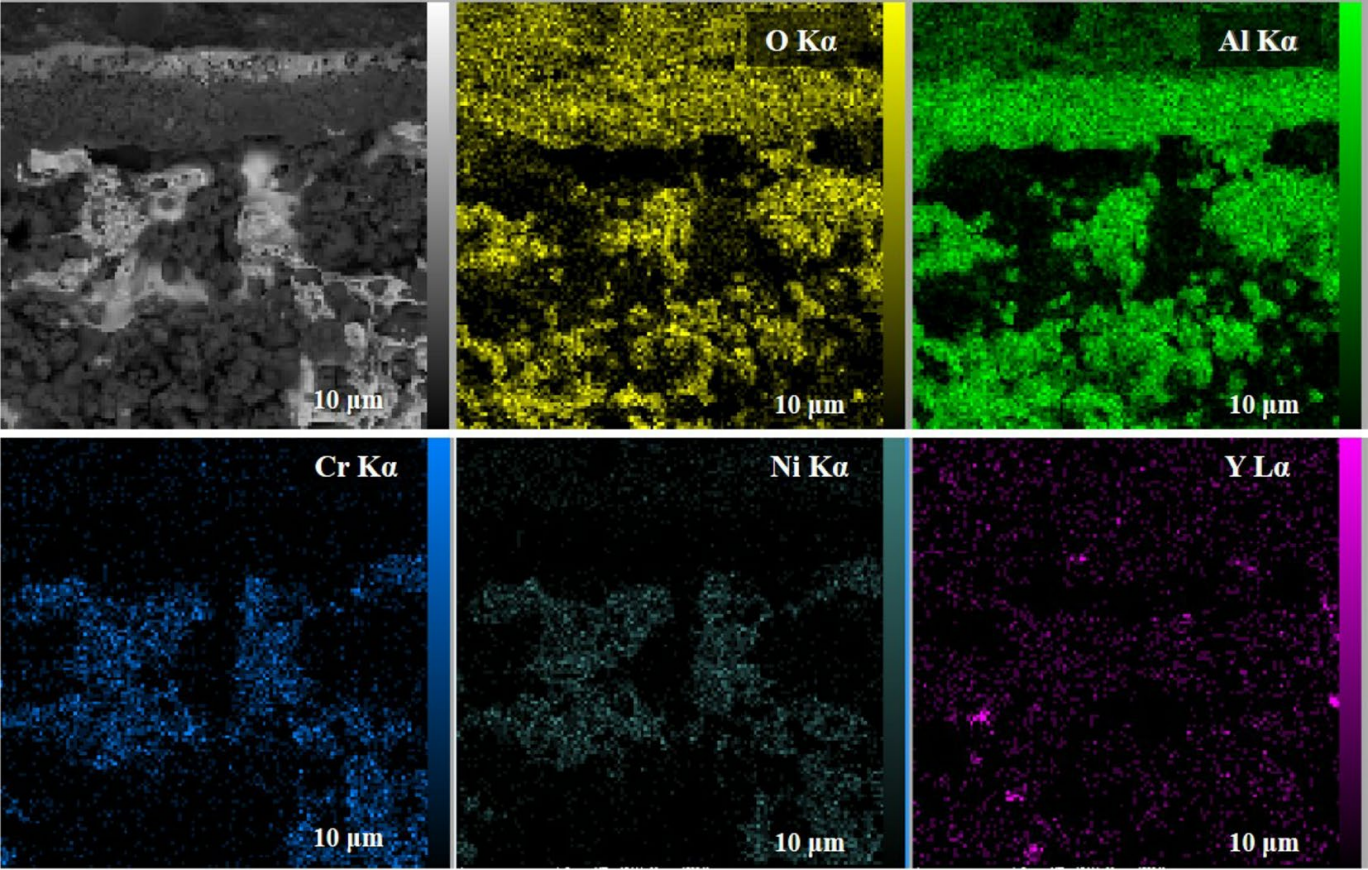
FESEM electron image and EDX elemental mapping for the nanocrystalline MCrAlY coating after long-term exposure at 1000 °C for 1000 h.

Figure 16 depicted the cross-sectional FESEM image as well as the EDX elemental mapping of the oxide layer formed on the nanocrystalline MCrAlY coating under the long-term exposure after 1000 h. According to the FESEM image, an adherent double-layered oxide scale with ~ 5 μm is formed on the coating surface. Furthermore, when looked at in more detail, no traces of oxide spallation/detachment and interfacial cracking were observed in the structure of the oxide scale. EDX elemental mapping also specified that the oxide layer is mainly composed of Al_2_O_3_ rather than spinel. This is attributed to the chemical decomposition of NiAl_2_O_4_ spinel to the Al_2_O_3_ during long-term exposure at 1000 °C up to 1000 h by the following reaction:

3 $${\text{NiAl}}_{{2}} {\text{O}}_{{4}} \to {\text{ NiO }} + {\text{ Al}}_{{2}} {\text{O}}_{{3}}$$

Besides that, the NiO monoxide phase can accordingly react with Al_2_O_3_ to form NiAl_2_O_4_ spinel during long-term oxidation testing^23,56,57^. Indeed, the monoxide NiO phase was transformed into spinel to increase the stability of the oxide in the early stages of oxidation. Otherwise, during prolonged oxidation at higher temperatures (greater than 900 °C), spinel oxide may decompose to stable Al_2_O_3_ oxide according to reaction 3.

In accordance with the research work performed by Lu et al.^58^, transient/mixed oxides and spinel can preferably form around the structural pores following the inadequate supply for Al ions. Whereas, in the regions around the pores, because of the large surface/volume ratio may cause high reactivity, so that Al is consumed more rapidly from the alloy and its concentration drops to values low enough to inhibit the formation of alumina and therefore allow the formation of transient/mixed oxides and spinel in the coating structure.

Therefore, the lower percentage of the structural porosities in the nanocrystalline MCrAlY coating obtained from 15 h milling powder feedstock is possible to inhibit the nucleation and development of fast-growing oxides (e.g., spinel) on the coating surface during the oxidation process. Consequently, the nanocrystalline MCrAlY coating has a greater oxidation resistance compared with the conventional coating in short- and long-term modes. This improvement is mainly attributed to the lower oxide growth rate consisting of higher volume rato of Al_2_O_3_ oxide and the low volume ratio of the brittle transient/mixed oxides in the nanocrystalline MCrAlY coatings.

**Figure 17:**
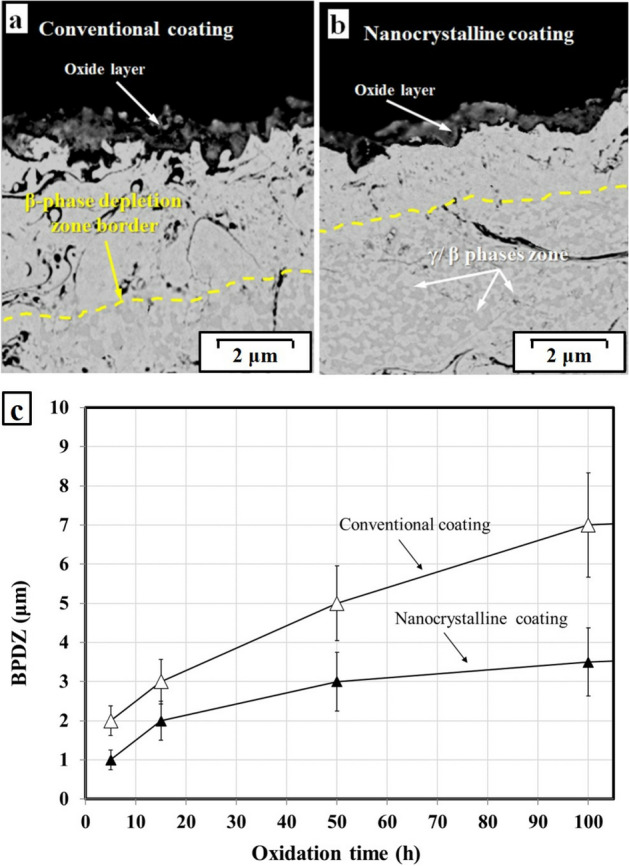
The BPDZ for the (**a**) conventional and (**b**) nanocrystalline MCrAlY coatings after isothermal oxidation at 1000 °C up to 100 h and (**c**) variations of BPDZ versus oxidation exposure time at 1000 °C up to 100 h.

During the short-term oxidation test, the overall amount of Al ions in the MCrAlY coating start to decrease due to the depletion of the Al-reservoir intermetallic NiAl β-phase. In this regard, the β-phase depletion zones (BPDZ) for the conventional and nanocrystalline MCrAlY coatings oxidized at 1000 °C after 100 h was presented in Fig. 17a,b. As following, the experimental measurements of the thickness of the BPDZ versus oxidation time for the conventional and nanocrystalline coatings are also presented in Fig. 17c.

As can be observed in Fig. 17a, the conventional coating has a wider BPDZ after 100 h of exposure compared to the nanocrystalline coating (see Fig. 17b). In fact, a higher percentage of Al ions from NiAl was consumed to create an Al_2_O_3_ oxide scale on the surface of the oxidized conventional MCrAlY coating. Conversely, for the nanocrystalline coating, a relatively narrow BPDZ was detected owing to the prohibition of Al-ion diffusion from β-phase by the higher volume ratio of structural defects and grain boundaries.

According to the experimental measurement for BPDZ (Fig. 17c), for all types of conventional and nanocrystalline coatings, the average width of BPDZ was rapidly increased at the first stages of the oxidation process due to the higher oxide scale growth rate (up to 15 h). Hence, a relatively broad BPDZ was formed especially for the conventional MCrAlY coating. Consequently, a higher value of β-phase is consumed to form the spinels and other transient oxides. Nevertheless, no significant BPDZ increment was detected in the range of 15–100 h, due to the reduction of oxidation rate and the formation of a continuous and protective Al_2_O_3_ scale on the coating surface. In contrast, a thinner BPDZ can be observed in the nanocrystalline coating. This behavior may be attributed to the barrier effect of the higher value of grain boundaries in the nanocrystalline coating structure against the outward diffusion of Al ions to the surface^59,60^.

#### Cyclic oxidation test

Table 5 listed the weight gain versus the number of thermal cycles for the conventional and nanocrystalline MCrAlY coatings that were conducted at 1100 °C under static air. In addition, the “n” index also has been calculated for both types of coatings after 100 cycles. As can be expected, during the early stages of the thermal cycling, the specific weight gain of the conventional and nanocrystalline coatings was monotonically increased following the increment of the number of thermal cycles up to 100. In addition, both conventional and nanocrystalline MCrAlY coatings followed the parabolic rate law under the thermal cycling at the early stages of the test (n index was 0.51 and 0.42 for the conventional and nanocrystalline coatings respectively). Moreover, when looked at in more detail, the overall rate of the weight gain during the early stages of the thermal cycling was relatively lower for the nanocrystalline MCrAlY coating. The specific weight gain of the coatings was concurrently started to decrease due to the increasing number of cycles more than 100. Subsequently, the weight loss of the coating caused by oxide spallation was started and continued up to 300 thermal cycles. In this regard, a higher drop rate was obtained for the conventional MCrAlY coating.

**Table 5:** Variation of the weight gain per unit area versus the number of oxidation cycles as well the n index (calculated after 100 cycles) for the conventional and nanocrystalline MCrAlY coatings under cyclic oxidation test at 1100 °C.

Number of cycles	Average weight gain per unit area (mg cm^−2^)	
	Conventional coating	Nanocrystalline coating
5	0.56	0.49
10	0.97	0.75
25	1.53	0.85
50	2.05	1.12
100	1.15	1.52
200	− 1.82	− 0.66
300	− 1.35	− 0.87
Oxidation behavior (up to 100 cycles)	Parabolic	Parabolic
n index	0.51	0.42

**Figure 18:**
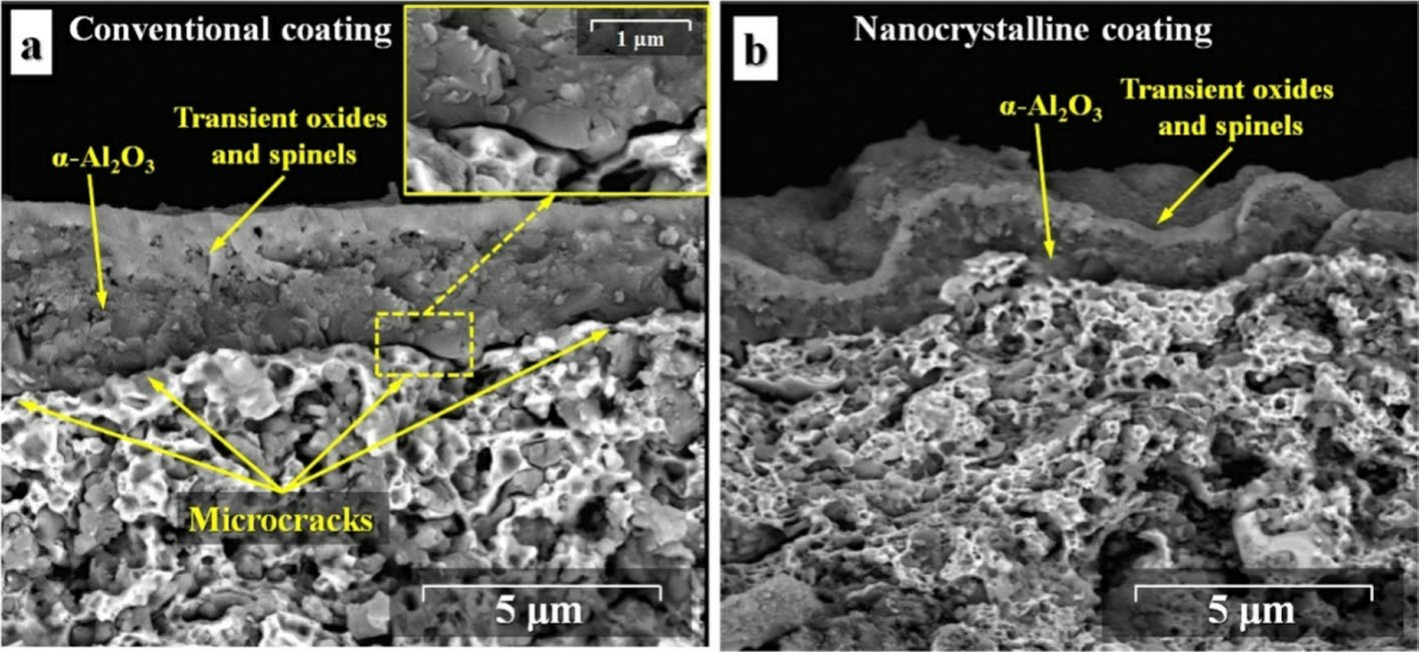
Cross-sectional FESEM image of the oxide scale formed on (**a**) conventional and (**b**) nanocrystalline MCrAlY coatings obtained by 15 h milled powder feedstock under thermal cycling test under 1100 °C after 300 cycles.

Figure 18 represents the freestanding conventional and nanocrystalline MCrAlY coatings after 300 thermal cycles at 1100 °C. As can be observed, internal oxide spots were formed into the microstructure of both coatings, but the volume ratio of the internal oxide regions for the conventional MCrAlY was greater than the nanocrystalline coating. According to Fig. 18a, a relatively thick oxide layer with a coarser morphology was developed in the conventional MCrAlY coating. This oxide scale is a continuous but irregular layer containing obvious defects, which indicate frequent spallation and re-formation of the new oxide scale during the thermal cycling test.

Among them, a fast-growing of the oxide scale was easily detectable on the surface of the as-received MCrAlY coating. These types of mixed oxides were preferably formed instead of alumina due to the prohibition of the Al-ions through the limited diffusion paths caused by the lower amount of surface grain boundaries in the as-received MCrAlY coating. Also, for the as-received coating, the microcracks were initiated and then propagated through the oxide/coating interface, correspondingly. Conversely, a thin and dense oxide scale was subsequently developed on the nanocrystalline MCrAlY coating surface. Nonetheless, no traces of interfacial cracks and coating spallation/detachment were observed in the oxide layer formed on the nanocrystalline coating after 300 cycles at 1100 °C (see Fig. 18b). The formation of a thin and continuous oxide scale in the nanocrystalline coating was attributed to its finer microstructure and the higher volume ratio of surface grain boundaries which can control the growth rate of the oxide scale after a prolonged thermal cycling test.

According to the observations by Unocic et al.^56,61^, a reason for increased oxide scale spallation from the MCrA1Y coating could be related to the higher content of C into the coating structure. With the presence of high C content, possible Cr-rich carbide formation at the coating/oxide scale interface was probably indicated to promote oxide scale spallation and cracking from the coating during the isothermal or cyclic oxidation process. Besides, according to the results obtained by Lu et al.^39^, the distribution of the fine-grained Y-rich phases into the nanocrystalline coating (even as ultrafine-grained Ni–Y or Y_2_O_3_ oxides) can prevent the oxide growth rate during high-temperature exposure of the coatings. Additionally, the greater thermal cycling stability of the nanocrystalline coating deposited by the nanostructured feedstock may be related to the creation of the oxide scale with a higher adhesion due to the precipitation of well-distributed Y in the coating structure. Following the results attained by Lu et al.^39,55^, the degree of the distribution of Y in the coating structure is attributed to the amount of SPD and subsequent cold welding during milling of the powder feedstock. The distribution of Y in the coating layer can also decrease the level of residual stress in the oxide scale formed on the coating surface. All of the above-mentioned results may cause to improve the oxide scale adhesion, decrease the possibility of the oxide detachment, and enhance the thermal cycling stability of the nanocrystalline coating compared to the conventional coating.

#### Oxide scale nucleation and growth mechanism

**Figure 19:**
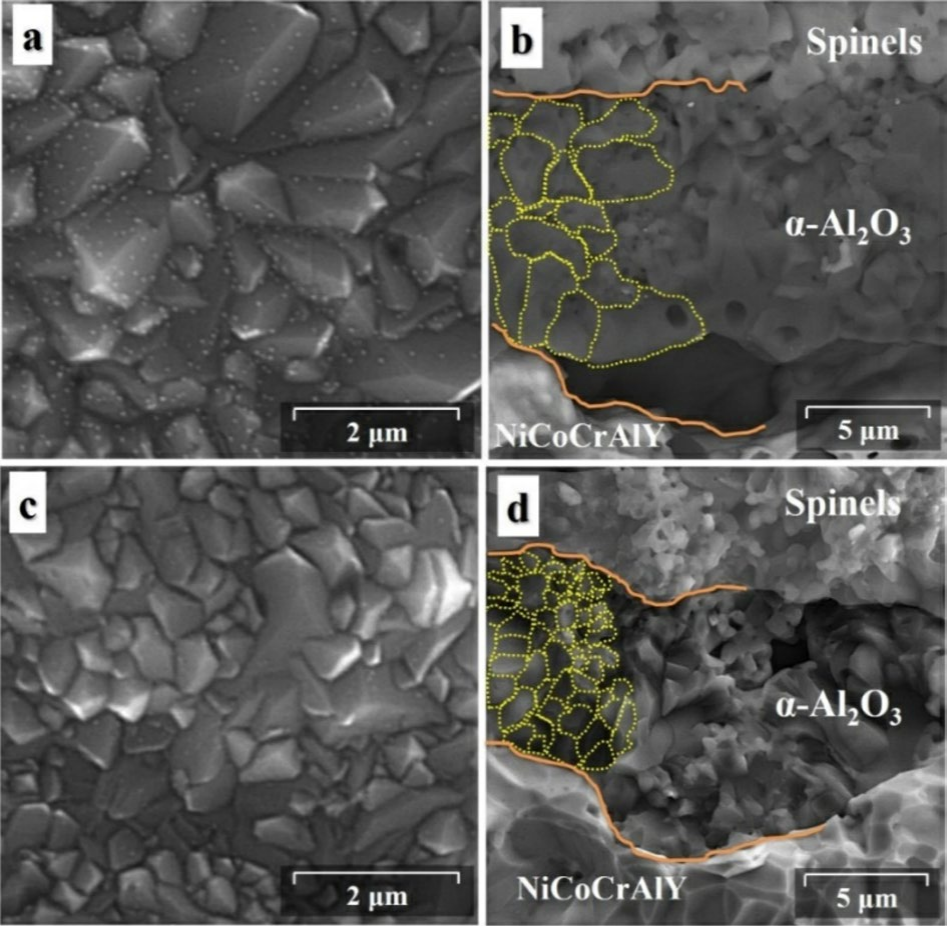
The top and the cross-sectional view of the morphology of the oxide scale formed on the conventional (**a**, **b**) and nanocrystalline (**c**, **d**) coatings after long-term oxidation at 1000 °C after 1000 h.

The morphology of the oxide scale formed on the coating surface has a direct effect on its growth rate during the long-term oxidation process. Figure 19 shows the top and the cross-sectional view of the morphology of the oxide scale formed on the surface of the conventional and nanocrystalline MCrAlY coating after the long-term oxidation process at 1000 °C for 1000 h. The observation of microporosities in the oxide layer for both oxidized conventional and nanocrystalline coatings could be due to the θ-Al_2_O_3_ to α-Al_2_O_3_ transformation, the solid-state formation of NiAl_2_O_4_ spinel from NiO monoxide and Al_2_O_3,_ or the evaporation of Cr-containing oxides as a form of CrO_3_ upon prolonged oxidation process at 1000 °C ^56^.

For the conventional coating, as can be seen from Fig. 19a, the oxide scale has a relatively coarser morphology compared with the nanocrystalline coating (Fig. 19c). Indeed, the initial fine-grain microstructure of the splats could yield a significant benefit. Namely, those surface grain boundaries between nano-sized crystallites and splats as well as in situ oxide phases could offer many nucleation sites for the formation of the Al_2_O_3_ oxide. Whereas, these defects can potentially act as heterogeneous nucleation sites for the formation of a continuous oxide scale on the coating surface^23^. As can be seen from Fig. 19b, a relatively thicker Al_2_O_3_ black oxide layer with a coarser morphology was formed on conventional MCrAlY due to its higher growth rate upon prolonged oxidation at 1000 °C after 1000 h. In this case, the average grain size of the oxide scale was estimated about ~ 3.14 μm. Conversely, a thinner Al_2_O_3_ black oxide was formed with a finer morphology (Fig. 19d) compared with the conventional coating. The average grain size of the oxide scale was obtained about ~ 0.85 μm. The finer grain morphology is mainly attributed to the lower oxide growth rate for the nanocrystalline coating at the same oxidation duration. Jedliński et al.^62^ also obtained the Al_2_O_3_ with fine-grained morphology and higher resistance to spallation by the addition of a small amount of Y into the FeCrAl coating composition.

For both conventional and nanocrystalline MCrAlY coatings, the overall concentration of Al ions is less than other metallic ions such as Ni. Therefore, during high-temperature exposure of the coatings (either as isothermal or cyclic modes), there are fewer Al-ions available to form the Al_2_O_3_ scale. On the contrary, other types of oxides (e.g., NiO or NiAl_2_O_4_) started to create in the early stages of the high-temperature oxidation. It is possible that the Al activity is not sufficient to develop pure Al_2_O_3_ on the surface of the MCrAlY coating. Therefore, after the formation of NiO, the NiAl_2_O_4_ spinel may also form at the early stages of the oxidation process. In this regard, Evans et al.^63^ indicated that the nucleation and growth of the Cr- or Ni-enriched oxides might be predominated for MCrAlY bondcoat in a typical TBC coated part at the beginning of the high-temperature service.

While in another viewpoint, more diffusion paths are accessible in typical HVOF-sprayed coatings. Whereas, the boundaries between splats are preferable paths to exterior Al diffusion towards the oxide layer. On the other hand, the atomic oxygens can also inter-diffuse in opposite directions through the coating layer from the mentioned paths to perform internal oxidation in the coating structure. The formation of internal oxide spots is also may attributed to the re-arrangement of pre-existing oxide inclusions using the diffusion process in the as-sprayed conventional and nanocrystalline coatings during long-time exposure at high-temperatures. As mentioned earlier, internal oxidation spots may occur through the coating structure by the chemical reaction of pre-dissolved oxygen arising from the HVOF process^20,26^.

According to Evans et al.^63^, the formation and growth of the oxide layer on the MCrAlY coatings mainly depend on the “diffusion cells” in the coating structure. Therefore, due to the higher affinity of Al-ions at temperatures more than 900 °C, the possibility of the formation of the stable Al_2_O_3_ is more than those NiO and NiAl_2_O_4_ spinel. Despite the nickel monoxide, the chromium oxide (Cr_2_O_3_) is often evaporated above 950 °C with the presence of relative air humidity^64^. As a summary, in the nanocrystalline MCrAlY coatings, following the results by Evans et al.^63^, the overall growth rate of the oxide layer was monotonically reduced owing to the surrounding of the surface by the protective Al_2_O_3_ oxide scale.

**Figure 20:**
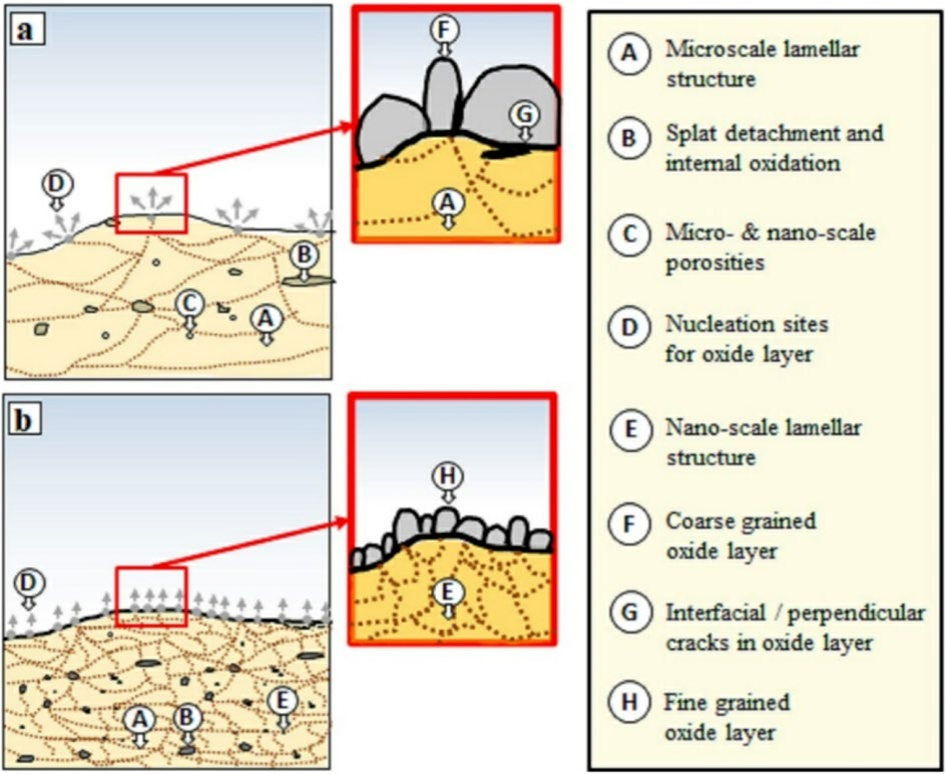
Schematic of the nucleation and growth of the oxide layer for the (**a**) conventional and (**b**) nanocrystalline MCrAlY coatings.

The comparative mechanism of the nucleation and growth of the oxide layer formed on both conventional and nanocrystalline MCrAlY coatings are presented in Fig. 20. According to this scheme, surface grain boundaries are the mainly preferable regions for the nucleation of the Al_2_O_3_ and the beginning of the oxidation process. For the case of the conventional coating (Fig. 20a), because of the coarse lamellar structure, there are limited nucleation sites for the oxide formation. Therefore, the oxide can grow with a higher thickness and coarser structure. Indeed, the thickening of the coarse-grained oxide has lower affected by the adjacent grains due to the limitation of nucleation zones. Among the formation of the thicker oxide layer, the residual stress may cause to initiate the interfacial/perpendicular cracks. These cracks may increase the tendency of the spallation of the oxide scale upon a prolonged oxidation process.

Conversely, as can be seen from Fig. 20b the nanocrystalline coating with a higher volume ratio of grain boundaries has a thinner oxide layer with a finer morphology. For this type of coating, regions for the oxide nucleation are relatively higher than that of conventional MCrAlY coating. Following the increase of the nucleation regions, the growth rate of each grain of the oxide is subsequently restricted by numerous adjacent grains. Therefore, the overall growth rate of the oxide formed on the nanocrystalline coating is lower than the conventional coating. As a consequence, following the lower oxide growth rate in nanocrystalline MCrAlY coating, the oxide scale was dense, more protective, and approximately free of crack.

In addition, due to the oxygen intake from the HVOF spraying process, internal oxide regions can form during high-temperature exposure for all types of conventional and nanocrystalline MCrAlY coatings. Subsequently, dissolved atomic oxygen can react with Al atoms to form internal oxidation. As can be seen from the schematics, more oxygen may absorb by nanocrystalline MCrAlY powder feedstock during HVOF owing to its agglomerated morphology. In contrast with the oxidized conventional MCrAlY coating under isothermal (see Fig. 15a) and cyclic oxidation process (Fig. 18a), for the nanocrystalline MCrAlY coating under both isothermal (see Fig. 15b) and cyclic (see Fig. 18b) oxidation testing, higher internal oxides are detected in the coating structure. This fact is attributed to easier oxygen interdiffusion from the oxidative atmosphere and higher soluble oxygen to react with Al ions resulting in the formation of the internal oxides into the coating structure.

## Conclusions

Based on this study, the structural and thermal properties of the nanocrystalline MCrAlY coating have been analyzed and the results were then compared with the conventional MCrAlY coating. Besides, the structural changes during isothermal and cyclic oxidation have been discussed in detail. Eventually, the main conclusions are presented as follows.

- Nanocrystalline MCrAlY powder feedstock was prepared via high-energy ball-milling after 15 h with a rotational speed of 300 rpm. The morphology of the as-milled powders was bulky-disk or agglomerated. Additionally, the nanocrystalline MCrAlY coating was also applied by the HVOF spraying technique.
- The nanocrystalline MCrAlY coating had a higher oxidation resistance (up to 32%) compared to the conventional coating due to the formation of a dense and slow-growing Al_2_O_3_ oxide layer. This oxide may act as a barrier layer to control the inward diffusion of oxygen and outward diffusion of metallic ions.
- Both conventional and nanocrystalline coatings follow parabolic rate law under short-term oxidation testing at 1000 °C up to 100 h and the oxide growth rate of all coatings is diffusion-controlled. Nevertheless, a sensible deviation from parabolic to sub-parabolic rate law has been observed for the nanocrystalline MCrAlY coating under the long-term oxidation.
- The nanocrystalline MCrAlY coating had a thinner BPDZ depth compared with the conventional coating at the oxidation time (up to 500 h) owing to the control of the outward diffusion of Al ions in the coating structure.
- Thermal cycling test results at 1100 °C indicated that nanocrystalline MCrAlY coating has greater thermal cycling stability than conventional coating because of its inhibition to initiate and growth of thermal-induced cracks and its resistance to oxide scale spallation during the thermal cycling test.
